# Informed Decision-making for Health Insurance Enrollment: Survey Study

**DOI:** 10.2196/27477

**Published:** 2021-08-12

**Authors:** Coralys M Colón-Morales, Wayne C W Giang, Michelle Alvarado

**Affiliations:** 1 Department of Industrial and Systems Engineering University of Florida Gainesville, FL United States

**Keywords:** health insurance, information, sources, survey, literacy

## Abstract

**Background:**

Health insurance enrollment is a difficult financial decision with large health impacts. Challenges such as low health insurance literacy and lack of knowledge about choosing a plan further complicate this decision-making process. Therefore, to support consumers in their choice of a health insurance plan, it is essential to understand how individuals go about making this decision.

**Objective:**

This study aims to understand the sources of information used by individuals to support their employer-provided health insurance enrollment decisions. It seeks to describe how individual descriptive factors lead to choosing a particular type of information source.

**Methods:**

An introduction was presented on health insurance plan selection and the sources of information used to support these decisions from the 1980s to the present. Subsequently, an electronic survey of 151 full-time faculty and staff members was conducted. The survey consisted of four sections: *demographics, sources of information, health insurance literacy,* and *technology acceptance*. Descriptive statistics were used to show the demographic characteristics of the 126 eligible respondents and to study the response behaviors in the remaining survey sections. Proportion data analysis was performed using the Cochran-Armitage trend test to understand the strength of the association between our variables and the types of sources used by the respondents.

**Results:**

In terms of demographics, most of the respondents were women (103/126, 81.7%), represented a small household (1-2 persons; 87/126, 69%), and used their insurance 3-12 times a year (52/126, 41.3%). They assessed themselves as having moderate to high health insurance literacy and high acceptance of technology. The most selected and top-ranked sources were *Official employer or state websites* and *Official Human Resources Virtual Benefits Counselor Alex*. From our data analysis, we found that the use of official primary sources was constant across age groups and health insurance use groups. Meanwhile, the use of friends or family as a primary source slightly decreased as age and use increased.

**Conclusions:**

In this exploratory study, we identified the main sources of health insurance information among full-time employees from a large state university and found that most of the respondents needed 2-3 sources to gather all the information that they desired. We also studied and identified the relationships between individual factors (such as age, gender, and literacy) and 2 dependent variables on the types of primary sources of information. We encountered several limitations, which will be addressed in future studies.

## Introduction

### Background

Enrolling in a health insurance plan is a complex decision that can have large health and financial impacts [1]. The decision is based on many factors (eg, premium costs, current health status, and the number of people covered) and directly affects future health care decisions such as choosing a provider or treatment. Therefore, informed decision-making is key for effective health insurance enrollment decisions. However, there are barriers to effective and informed enrollment decisions. Poor understanding of basic health insurance terminology (eg, deductible, premium, and copay) is the main barrier [2] because only 4% of the US population accurately understands such terms [3]. Lack of knowledge about how to choose a plan is another barrier to efficient enrollment decisions. Thus, consumers can benefit from specific information regarding health insurance literacy, available plans, and guidance on how to choose a plan based on the needs of individuals. Informed decision-making requires access to, and understanding of, useful information that can support the decision-making process. Over the last few decades, the proliferation of digital resources has changed how health information is accessed, but little research has examined how these changes have influenced health insurance decision-making. Therefore, the first aim of this paper is to understand the history of health insurance enrollment decision research and how decision-making has changed with the development of new digital technologies.

Currently, there are many sources of health insurance information used by individuals; however, these sources can sometimes lack accessibility, accuracy, or completeness of information, which can lead individuals to use more than 1 source. For our study, we considered the following types of sources: meetings with benefits representatives, printed material from health insurance providers, friends and family, websites, and virtual benefits counselors (VBCs). A VBC is a type of digital decision aid that mimics one-on-one conversations with a human resources (HR) benefits counselor using a conversational digital interface. VBCs provide personalized guidance in the decision process by contrasting different health insurance plans while providing increased access to health insurance literacy. Many employers have created customized VBCs to provide decision support to their employees. The VBC in this study refers to the official HR VBC Alex (Jellyvision Labs Inc) [4].

As individuals choose the source of information that they wish to use, their selection can vary depending on their goals, preferences, and knowledge about health insurance [5]. Similarly, a person’s attitude toward technology could also have an impact on the source of information that they choose to use as support for their enrollment decision [6,7]. Therefore, the second aim of this paper is to explore where current consumers are searching for information to support their employer-provided health insurance decisions. To achieve this aim, we created and used a survey to gain insight into the current sources of information used by employees of a large state university. This paper presents the pilot study for this project; therefore, the work shown here is exploratory in nature.

To bridge the gap in our understanding of how health insurance consumers achieve informed enrollment decisions, it is essential to understand how individuals go about making these decisions and what information they use to support their choice. During the 1980s and the 1990s, with the rise of health maintenance organizations in the health insurance market, extensive research was conducted on the information factors that influence health plan selection and on how individuals use this information to decide from the options available to them. For example, Scanlon et al [5] compiled and analyzed numerous studies. They identified and categorized a set of variables to rationalize the variation in health plan choices. The primary variables referred to health plan characteristics, which included costs, provider restrictions, different types of coverage and benefits, quality, and convenience. The secondary variables referred to consumer demographics and other characteristics such as health and economic status. Although the research showed that the primary variables, particularly price, influenced the plan choice, many studies also showed that, depending on the age, gender, or health status, groups can have distinct patterns of enrolling in specific health plans [8-10]. Therefore, the distinction between the primary and secondary variables and the interactions between them are important for understanding plan selection bias and the probability of a consumer enrolling in a particular plan. Prominent empirical studies during the 1980s and 1990s used (1) modeling consumer choice under uncertainty [9,11], (2) conditional choice models that supported the estimation of the trade-off between price and other health plan characteristics included in the model [12], or (3) probit or logit analysis, which allowed modeling the probability of enrolling in a plan as a function of price and other plan features [13].

Studies also sought to understand the ideal types of information that consumers preferred when making health insurance enrollment decisions, and Edgman-Levitan and Cleary [14] concluded that consumers need comparative data on the various plans; trade-off evaluations among access, cost, and quality; and methods to determine their out-of-pocket costs based on their health status. In contrast, Isaacs [15] pointed out the need for information on the quality of primary care physicians and specialists, the range of services covered, pre-existing condition exclusions, and consumer ratings of hospitals and physicians. Similarly, Tumlinson et al [16] showed that cost, price, benefits, availability, and quality of providers are essential when comparing plans and making an informed decision. However, across these studies, there was an acknowledgment of the challenges faced when creating ways to provide and present this information to support consumer understanding. This shows that the process of choosing a health insurance plan is complex and typically leads individuals to make poor decisions [17].

Various types and sources of health insurance information were examined during the 1980s and 1990s; for example, digital methods of presenting information to consumers that present different *layers* of information depending on individual interests. As another example, consumer report cards were a popular mechanism for sharing health plan performance measures through consumer ratings and insights into the available health plans. Although these methods have the potential to provide quality information, it is important to have a thorough comprehension of this information [18]. However, Gibbs et al [19] discussed that consumer satisfaction measures need to be carefully chosen and presented to communicate meaningful information and appeal to specific consumer needs. Compared with report cards, information sources that are impartial to health insurance companies were preferred by the participants in this study; therefore, they valued the input from friends and family. The findings of the study by Isaacs [15] revealed similar preferences, along with trust in health plan information received from the primary physician.

With the diffusion of the internet during the late 1990s and the early 2000s, websites became a source of health and medical information [20], especially among younger consumers [21,22], consumers with chronic conditions, and those who face barriers when accessing health care [23]. Therefore, many scholars analyzed the internet’s impact on consumers’ search for health information. Studies showed that more people were searching the web for medical information before talking to their doctors [24], and federal websites were widely considered trustworthy sources of health information [25]. In fact, the results of a study by Rains [26] showed that lack of trust in the traditional sources of information (eg, mass media and one’s health care provider) was associated with the increased use of websites as a primary information source. However, when it was difficult to find or understand health information on the web, there was less trust in it [27]. When comparing the primary sources for health information among individuals, Dutta-Bergman [28] reported that the internet, newspapers or magazines, and family or friends were the primary sources of health-conscious individuals. These studies focused on the effect of the internet on the search for health information in general and provided a baseline for the main categories of primary sources of information considered in this paper: official sources such as federal websites, internet-based sources, and trusted individuals such as friends and family.

However, few studies during the period between the late 1990s and the early 2000s focused on sources of health insurance information. A study by Mark and Coffey [29] evaluated the available sources of health plan information for consumers. They considered consumer report cards and report filings from state insurance departments, health plan websites, the US Census, independent organizations, and a national survey on health care use. Although each source had its strengths, the authors concluded that all sources would benefit from more practical and cost-effective methods for providing information. It is also important to look at the decisions of employees regarding employer-provided insurance because there are other factors that affect their decisions [30]. A later study by Oetjen et al [31] focused on government employees’ access to and use of three health plan information sources: printed information from the state, printed information from the health plans, and web-based information. In this 2003 study, the most accessed and used source was printed information from the health plans, followed by printed information from the state and, finally, web-based information, with 34% of the participants using all 3 sources.

With the passing of the Affordable Care Act in 2010, more focus was given to understanding the complexity of choosing a health insurance plan because the act sought to expand coverage, and new decision support tools began to be developed. This became critical to ensuring enrollment success [32,33]. Studies found that plan choice complexity emerges from the wide variation in health costs and other plan features such as network size, service coverage, and reputation on processing claims [2], as well as the level of engagement from consumers [34]. Therefore, to make an informed decision, consumers must project their health expenses and subsequently evaluate the trade-off among the plan’s health costs. However, this is still a challenge for most people because only 4% of the US population understands basic health insurance terminology [3]. In fact, Hero et al [35] showed that among people with low health insurance literacy, at least 54% had difficulty finding the best or most affordable plan, and at least 48% had fair or poor overall experience when choosing their plan. These findings suggest that disparities in the ability to access and understand health insurance information may be a reason why different demographics may have differing plan selections [36].

Thus, to better support health insurance decision-making, different digital decision support tools such as cost estimators, quality ratings, provider lookup, drug lookup, and pop-up definitions started to become more available to consumers. These digital tools also allowed for the sorting and filtering of information by individual characteristics, a task that was much more difficult with printed media. However, in the beginning, these aids were missing from most federal and some state-based websites [37]. In fact, Vardell [38] points out the need for consumers to seek various sources of information (2.8 sources, on average) to fully assess their options. As studies began to highlight the importance of decision aids [39-41], these tools became the standard for informed consumer choice when the information was accurate and understood by consumers. Later studies have also shown a greater adoption of some decision support tools by websites for private and public health insurance marketplaces [42,43], a trend that is likely to continue.

The increased availability of decision aids led to the development of new tools that combine multiple methods for supporting enrollment decisions (eg, cost estimation, provider lookup, and definition of health insurance terms). One example is the Show Me My Health Plans (SMHP) tool, built to provide information about the Affordable Care Act marketplace in Missouri [44-46]. The SMHP tool contains simplified health insurance information (it uses plain language and graphics) for educational purposes, a cost-estimating component, an assessment of plan feature preferences, and plan recommendations. The SMHP decision aid improved the health insurance selection quality by improving decision self-efficacy, health insurance literacy, and confidence in plan choice compared with the health care government website [45]. More recently, companies have also developed aids that walk individuals through the enrollment decision-making process. VBCs are an implementation of these aids that have been used by HR departments to assist with employee plan selection. The continued development of these decision aids is likely to be of benefit to health insurance consumers, but it is still not known if consumers are adopting and preferring these tools in comparison with other sources of health insurance information.

### Objective

As shown in this study, health insurance enrollment choices are complex, multifactorial decisions that require access to different types of information. Although some studies have examined the sources of information that individuals access, the recent availability of new tools such as the SMHP tool and VBCs may have changed the landscape of information that users seek. These issues are of particular interest because employer HR departments are now recommending the use of these tools to their employees as a means of helping to support informed decision-making, especially because 56% of Americans receive their coverage from their employer [47]. Thus, a web-based survey of employees at a large state university was used to understand the sources of information used to support their health insurance enrollment decisions and to study the factors that led them to the sources that they use.

## Methods

### Health Insurance Information Sources Survey

The Health Insurance Information Sources Survey (HIISS) is a web-based survey containing 27 questions in total and takes approximately 5 minutes to complete on Qualtrics (Qualtrics XM). A sample of the HIISS questions is shown in Table 1, along with their respective response types and options. The full survey questions are available in Multimedia Appendix 1. The HIISS survey consists of four sections:

1. *Demographics and employment status*: The questions in this section are mostly categorical (either nominal or ordinal) and were all author-created. It is important to note that the age category of 55-66 years has a cutoff of 66 years because this is the social security full-benefits retirement age.
2. *Sources of health insurance information used*: This section contains only the 2 questions presented in Table 1. Both questions were author-created.
3. *Health insurance literacy*: Four questions were selected from the Health Insurance Literacy Measure (HILM) developed by Paez et al [48,49] (Table 1, section 3). This measure assesses 2 categories of health insurance literacy (selecting health insurance and using health insurance), each addressing 2 dimensions (confidence choosing and comparing plans in the first category and confidence using and being proactive in the second category). The 4 questions selected were each taken from a different category and dimension. In these scales, 1 represented not confident or not likely at all, whereas 7 represented extremely confident or likely.
4. *Technology acceptance and experience with virtual chatbots and agents*: In this section, the first 3 questions about technology acceptance were obtained from Reimer et al [50-53] and have scaled responses from 1 to 10, with 1 being very inexperienced or distrustful and 10 being very experienced or trustful. The other questions related to experience with virtual chatbots and agents were author created.

The HIISS was designed to gain insights into where employees are looking for health insurance information as well as the respondents’ demographic information, health insurance literacy, and interactions with technology. The questionnaire focused on six main sources of health insurance inspired by the background: official employer or state information, an official HR VBC system, friends or family, other nonofficial websites or resources (with space to write in), official HR in-person benefits counselors, and other in-person resources (with space to write in). It is important to note that the HR department at the employer had recommended the use of a VBC for enrollment decisions starting in 2017, which may have contributed to this greater awareness of this new type of technology. All data collected were confidential, and the study was approved by the local institutional review board (IRB #201900070).

**Table 1:** Health Insurance Information Sources Survey questions.

Questions			Response options
**Section 1: demographics**			
	For your last enrollment period, were you responsible, or did you share responsibility, in making health insurance decisions within your household? (nominal response)	Yes, I was primarily responsible Yes, I shared responsibility No, I did not share responsibility	
	What is your age? (ordinal response)	18-24 years old 25-34 years old 35-44 years old 45-54 years old 55-66 years old 67 plus years old Prefer not to say	
	Which of these best describes your gender? (nominal response)	Male Female Other Prefer not to say	
	Which of these best describes your marital status? (nominal response)	Single Married or domestic partnership Prefer not to say	
	Do you have dependents beside a spouse or partner? (nominal response)	Yes No Prefer not to say	
	How large is your household? (ordinal response)	1 to 2 3 to 5 6 or more	
	How often do you or your dependents use your health insurance? (ordinal response)	Never Less than 3 times a year 3 to 12 times a year 13 to 24 times a year More than 24 times a year Prefer not to say	
**Section 2: sources of information**			
	Where do you find information about health insurance plans? (Select all that apply)	Human Resources’ Alex, an online, virtual benefits counselor Other official or state website Other online websites or resources (eg, Google, government health care website; please indicate the website or resource) Human Resources’ in-person benefits counselor Friends or family Other in-person resources (please indicate the resources)	
	Please rank the sources of information that you use for health insurance enrollment decisions from most important (1) to least important. (Please select one ranking per source)	Each source from the question above is listed with 6 possible rankings to the right of each	
**Section 3: health insurance literacy (ordinal responses)**			
	(a) How confident would you feel that you understand health insurance terms (eg, copay, deductible, co-insurance, premium)?	7-point Likert scale, with 1 being *not confident at all* and 7 being *extremely confident*	
	(b) When comparing health plans, how confident are you in understanding what needs to be paid for emergency department visits?	7-point Likert scale, with 1 being *not confident at all* and 7 being *extremely confident*	
	(c) How confident are you in knowing what is and is not covered before you receive a health care service?	7-point Likert scale, with 1 being *not confident at all* and 7 being *extremely confident*	
	(d) When using your health insurance plan, how likely are you to find out if a doctor is in-network before you see him/her?	7-point Likert scale, with 1 being *not likely at all* and 7 being *extremely likely*	
**Section 4: technology acceptance**			
	(a) How would you rate your level of experience with technology (eg, cell phones, automatic teller machines, digital cameras, computers, etc)? (ordinal response)	A scale of 1-10, with 1 being *very inexperienced* and 10 being *very experienced*	
	(b) Some people prefer to avoid new technologies as long as possible while others like to try them out as soon as they become available. In general, how would you rate yourself as being an avoider or an early adopter of new technology? (ordinal response)	A scale of 1-10, with 1 being *avoid as long as possible* and 10 being *try as soon as possible*	
	(c) How would you rate your overall level of trust in technology? (ordinal response)	A scale of 1-10, with 1 being *very distrustful* and 10 being *very trustful*	
	Have you ever interacted with a “virtual agent,” “chatbot,” or “virtual rep” when interacting with a website or web service? Virtual agents provide automated customer service using a conversational interface. (nominal response)	Yes, multiple times Yes, I have tried them No, I have not tried them Not sure	
	How would you rate the usefulness of the virtual agents you’ve interacted with in the past? (ordinal response)	A scale of 1-10, with 1 being *not useful at all* and 10 being *extremely useful*	
	How would you rate the ease of using the virtual agents you’ve interacted with in the past? (ordinal response)	A scale of 1-10, with 1 being *not easy to use at all* and 10 being *extremely easy to use*	

### Recruitment

The HIISS was distributed to employees at a large state university through emails and flyers through academic and service departments, as well as through an HR newsletter sent to more than 31,000 employees. Given that this is an exploratory study, we allowed for snowball sampling to occur. Only qualifying respondents were included in the final data set. The inclusion criteria were as follows: (1) full-time employment (measured in full-time equivalents) at the university that qualified for health benefits and (2) being the primary decision-maker or sharing decision-making responsibility in the household for health care. We estimate that approximately 15,500 members are full-time staff or faculty members satisfying inclusion criterion 1.

### Statistical Analysis

We present descriptive statistics on the demographic variables, HILM scores, technology acceptance scores, the sources of information selected, and the source ranks assigned by the respondents. This allowed for an understanding of the sources of health insurance information used by employees to enroll in health plans.

As the respondents ranked their sources of information in order of importance, their highest-ranked source was considered their primary source of information. The response or dependent variables in our analysis centered on whether the primary source of information was an official source as opposed to a nonofficial source, as well as whether it consisted of friends or family. These binary factors were identified as important characteristics of the information sources in our background [25,28] and are therefore the main factors of interest in this study. Therefore, the 6 source options provided in the survey were classified as either official or nonofficial sources of information and as friends or family or not friends or family. Under official sources, we classified official employer or state websites, the official HR VBC system, and official HR in-person benefits counselors. Under nonofficial sources, we placed friends or family, other nonofficial websites or resources, and other in-person resources. Although there exist external websites (eg, government health care website) that contain validated general health insurance information and other helpful decision aids, they do not provide information on these specific employer-provided health insurance plans. Therefore, they were considered nonofficial sources of information for the scope of this study. Similarly, under not friends or family, we considered all the listed sources except friends or family.

This categorization allowed for the creation of two binary variables: (1) official (true) and nonofficial (false) primary sources and (2) friends or family (true) and not friends or family (false) as primary sources. Given that all our collected data are categorical variables (mostly ordinal) and that these primary source variables are binary, we required an association test for these specific types of variables. Ultimately, we decided upon the Cochran-Armitage trend test. The goal of these tests was to understand whether a relationship or a trend exists between our binary primary source—dependent variables—and our demographic variables, HILM scores, and technology acceptance scores. With these tests, we sought to identify whether there are respondents’ characteristics that drive their primary source decision. The Cochran-Armitage test is used to test whether there is a linear trend when the explanatory variable is ordinal and the response is binary. It assesses whether there is a monotonically increasing or decreasing trend in the proportions of the response Y over the ordered categories X. This test is applied to data in the form of an *r*×2 contingency table, with *r*>2. The null hypothesis is that no trend exists, which means that the proportions for all levels of the explanatory variable X are the same. On considering a 95% CI values, P<.05 indicates that an association or trend exists for the binary response Y over the categorical variable X. In the case of the demographic factor gender, which is a nominal variable, the association with our 2 binary responses was measured using the φ coefficient of correlation.

## Results

### Demographic Factors

Of the 151 individuals who responded to the HIISS, 126 (83.4%) were included in the final data set. Of the 25 participants who were excluded, 4 (16%) did not complete the survey, 4 (16%) did not share responsibility in making health insurance decisions within their household, and 17 (68%) had a current employment status of less than 0.75 full-time equivalent. Table 2 summarizes the respondents’ demographic information. Our sample comprised mostly women (103/126, 81.7%), with greater participation from the age groups 25-34 years and 55-66 years. In addition, most of the respondents (79/126, 62.7%) were married or in a domestic relationship and lived in households of 1-2 persons (87/126, 69%). Table 2 also shows how often the respondents’ households used their health insurance over the course of a year. Most commonly, the respondents used their insurance 3-12 times a year, as indicated by 41.3% (52/126) of the respondents.

Using Spearman correlation tests [54], Table 3 presents an upper-triangular correlation matrix (ie, there are only values above the diagonal) among the ordinal demographic factor variables and the health insurance use variable. As expected, the number of dependents and household size were positively correlated (ρ=0.81). Furthermore, household size was correlated (ρ=0.32) with higher health insurance use. The results found no evidence that age was associated with an increase in health insurance use.

**Table 2:** Respondents’ demographics (N=126).

Demographics			Health insurance use, n (%)											Total (N=126), n (%)
			Never (n=12)		Fewer than 3 times a year (n=19)		3-12 times a year (n=52)		13-24 times a year (n=26)		More than 24 times a year (n=17)			
**Age (years)**														
	18-24	0 (0)		2 (10.5)		3 (5.8)		1 (3.8)		0 (0)		6 (4.7)		
	25-34	7 (58.3)		3 (15.8)		19 (36.5)		6 (23.1)		3 (17.6)		38 (30.2)		
	35-44	1 (8.3)		4 (21.1)		11 (21.1)		6 (23.1)		2 (11.8)		24 (19)		
	45-54	1 (8.3)		3 (15.8)		9 (17.3)		7 (26.9)		4 (23.5)		24 (19)		
	55-66	3 (25)		6 (31.6)		10 (19.2)		6 (23.1)		8 (47.1)		33 (26.2)		
	≥67	0 (0)		1 (5.3)		0 (0)		0 (0)		0 (0)		1 (0.8)		
**Gender**														
	Female	9 (75)		14 (73.7)		46 (88.5)		21 (80.1)		13 (76.5)		103 (81.8)		
	Male	3 (25)		4 (21.1)		5 (9.61)		5 (19.2)		4 (23.5)		21 (16.7)		
	Other	0 (0)		0 (0)		1 (1.9)		0 (0)		0 (0)		1 (0.8)		
	Prefer not to say	0 (0)		1 (5.3)		0 (0)		0 (0)		0 (0)		1 (0.8)		
**Marital status**														
	Single	10 (83.3)		7 (36.8)		19 (36.5)		8 (30.1)		1 (5.9)		45 (35.7)		
	Married or domestic partnership	2 (16.7)		10 (52.6)		33 (63.5)		18 (69.2)		16 (94.1)		79 (62.7)		
	Prefer not to say	0 (0)		2 (10.5)		0 (0)		0 (0)		0 (0)		2 (1.6)		
**Household size (persons)**														
	1-2	11 (91.7)		16 (84.2)		38		15 (57.7)		7 (41.2)		87 (69)		
	3-5	1 (8.3)		3 (15.8)		14		11 (42.3)		10 (58.8)		39 (30.9)		

**Table 3:** Demographic correlation matrix.

Correlation matrix	Age (years)	Household size	Health insurance use
Age (years)	1	0.12	0.14
Household size	—^a^	1	0.32^b^
Health insurance use	—	—	1

^a^Not applicable.

^b^Significant correlations at α=.05.

### HILM and Technology Acceptance

**Figure 1:**
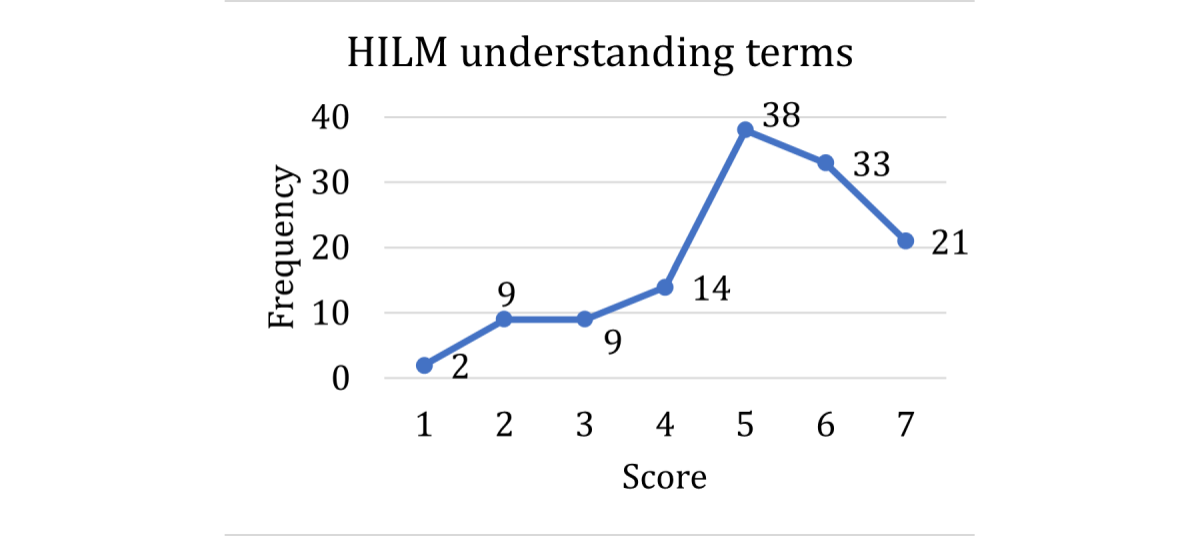
Scores for Health Insurance Literacy Measure in understanding terms. HILM: Health Insurance Literacy Measure.

**Figure 2:**
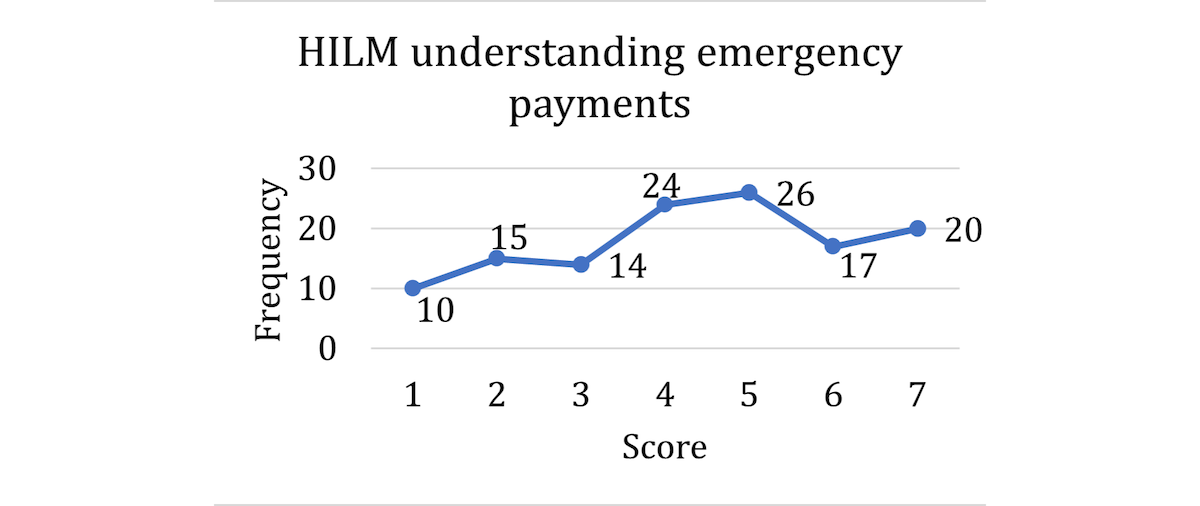
Scores for Health Insurance Literacy Measure in understanding emergency payments. HILM: Health Insurance Literacy Measure.

**Figure 3:**
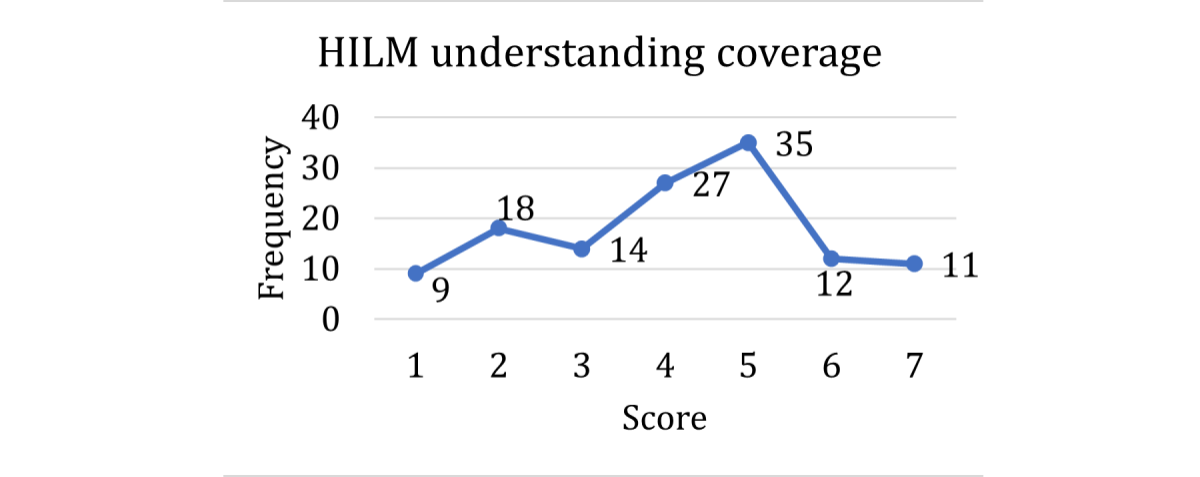
Scores for Health Insurance Literacy Measure in understanding coverage. HILM: Health Insurance Literacy Measure.

**Figure 4:**
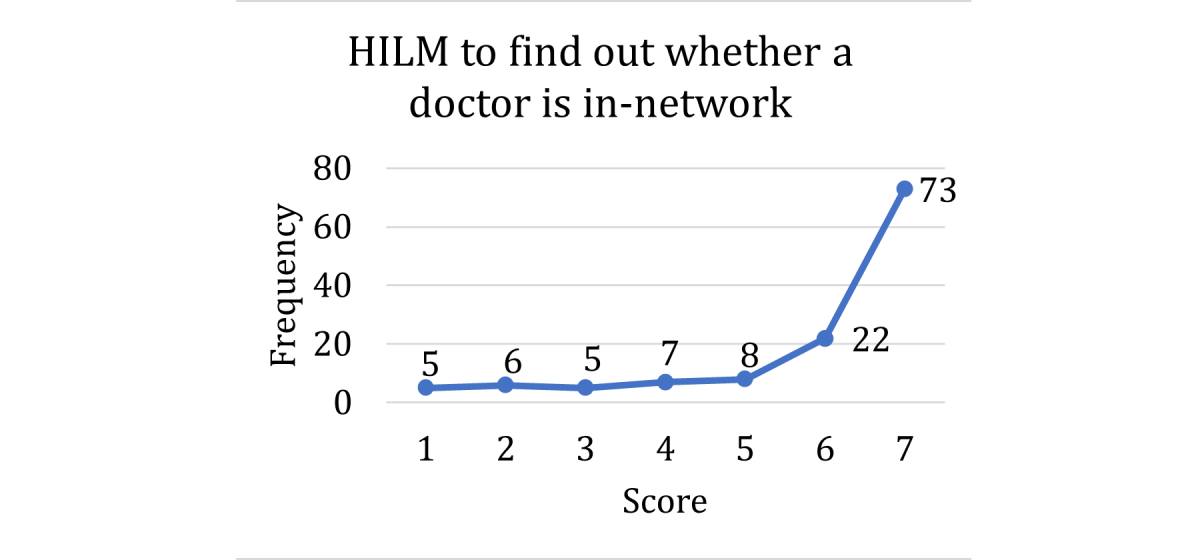
Scores for Health Insurance Literacy Measure to find out whether a doctor is in-network. HILM: Health Insurance Literacy Measure.

The score distributions for the HILM and technology acceptance are shown in Figures 1-4, with higher scores representing higher self-reported confidence or ability in that dimension of health literacy. A score of 5 was the mode among the questions on understanding terms (Figure 1), understanding emergency payments (Figure 2), and understanding coverage (Figure 3). For the question on understanding terms, there was a tendency toward a higher score (≥5), whereas for the questions on understanding emergency payments and understanding coverage, the score frequencies were balanced throughout the scale. However, the question on finding out whether a doctor is in-network (Figure 4) had a different response behavior, with 57.9% (73/126) of the respondents responding that they were extremely likely to find out whether a doctor is in-network before they see them.

**Figure 5:**
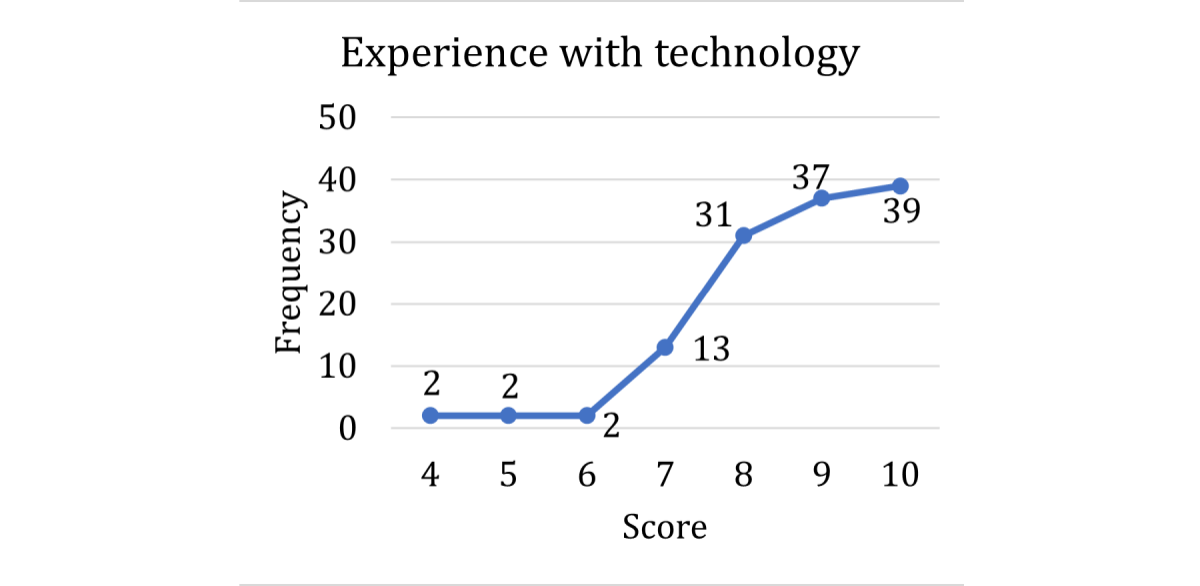
Scores for the self-reported technology experience question.

**Figure 6:**
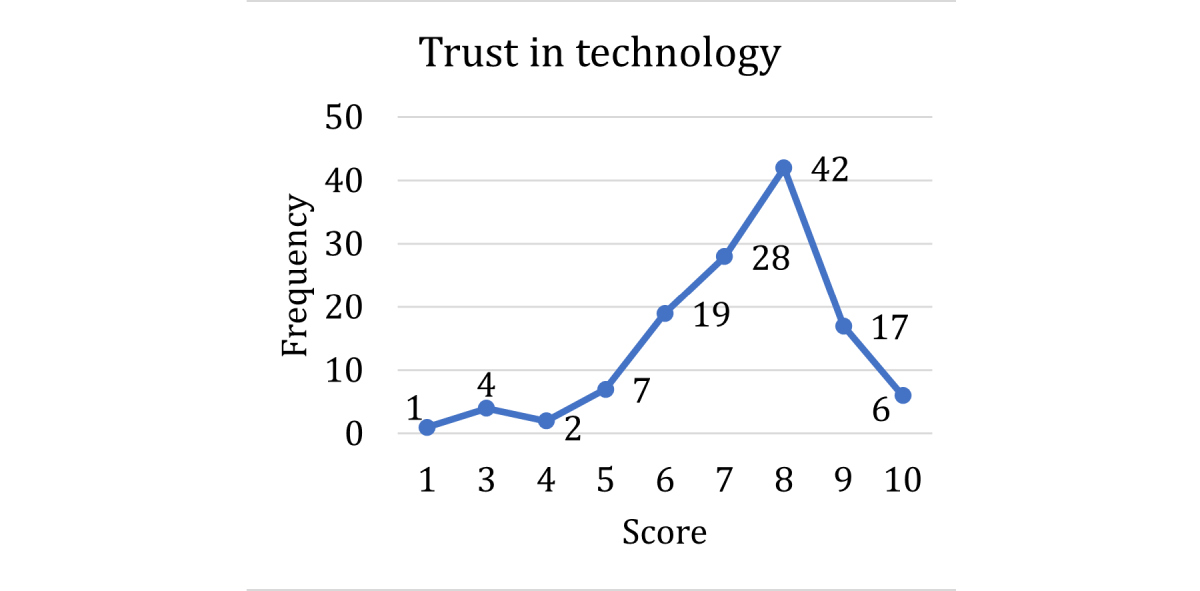
Scores for the self-reported technology trust question.

**Figure 7:**
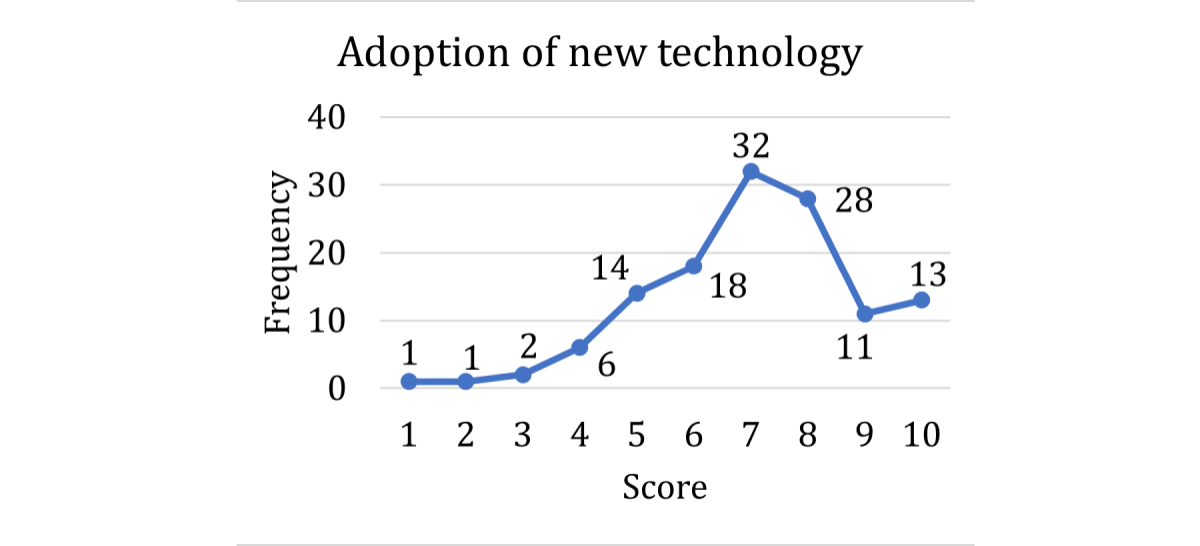
Scores for the self-reported adoption of new technology question.

The respondents’ technology acceptance and experience with virtual agents were also assessed (Table 1, section 4). All respondents had high levels of experience with technology; in fact, not a single response scored lower than 4, and most of the respondents rated their experience with technology as either 9 or 10 (Figure 5). For the most part, both trust in technology (Figure 6) and adoption of new technologies (Figure 7) were also high across the sample. Overall, our respondents—full-time employees at a large state university—had very high technology acceptance.

The respondents in this sample were also largely familiar with virtual agents, with only 15.9% (20/126) stating that they had never used (or were not sure if they had used) a virtual agent. The respondents were also asked to rate the usefulness of virtual agents on a 10-point scale. Of the 126 respondents, 44 (34.9%) had tried virtual agents, and the average reported usefulness was 4.72 (SD 1.92; median 5), and among the 65 (51.6%) respondents who had used them multiple times, the average usefulness was 6.51 (SD 1.99; median 7). Of note, the perceived usefulness among people who had used these agents multiple times was higher than among those who have only tried them.

### Sources of Information

The respondents were also asked where they found information regarding their health insurance plans. They were presented with the 6 options shown in Table 4 and asked to select all sources of information that they had used. On average, the respondents selected 2-3 sources of information, none selected all 6 options, and only 2 selected 5 sources of information (Table 5). The official employer or state websites were the most selected source (96/126, 76.2%), followed by official HR VBC Alex (76/126, 60.3%), both being digital and official sources of information. The third most selected source was friends or family with (58/126, 46%) a nonofficial and nondigital source. These results showed that most respondents made use of more than 1 source of information.

**Table 4:** Number of respondents who selected each source as an information source for health insurance plans (N=126).

Source of information	Respondents, n (%)
Official employer or state websites	96 (76.2)
Official HR^a^ VBC^b^ Alex	75 (59.5)
Friends or family	58 (46)
Other websites or resources (eg, Google and health care government website)	28 (22.2)
Official HR in-person benefits counselors	23 (18.2)
Other in-person resources	12 (9.5)

^a^HR: human resources.

^b^VBC: virtual benefits counselor.

**Table 5:** Number of sources respondents use for health insurance plan information (N=126).

Number of sources	Frequency, n (%)
1	25 (19.8)
2	50 (39.7)
3	39 (30.9)
4	10 (7.9)
5	2 (1.6)
6	0 (0)

The respondents were also asked to rank their selected sources in order of importance (Table 6; see the graph in the Multimedia Appendix 2 for a better visual understanding of Table 6). The official employer or state websites were ranked first by 49.2% (54/126) of the respondents, whereas the official HR VBC Alex and friends or family were ranked first by 21.4% (27/126) of the respondents. These results suggest that the official employer or state websites were the most used and preferred source of information. In contrast, the VBC decision aid seemed to be a supplementary resource because it had more second-place votes than first-place votes. Finally, although fewer respondents made use of friends or family, the most common rank that this source received was 1 (27/58, 47%). This suggests that there is a strong preference for family or friends among the persons who make use of this source.

**Table 6:** Ranking occurrences per source.

Source	Rank 1	Rank 2	Rank 3	Rank 4	Rank 5	Rank 6
UF^a^ human resources’ VBC^b^ Alex	27	32	10	5	0	0
Other official UF or state of Florida website	54	25	11	5	0	0
Other websites or web-based resources (eg, Google and health care government website)	9	10	9	0	0	0
UF human resources’ in-person benefits counselors	5	8	8	1	1	0
Friends or family	27	18	11	1	1	0
Other in-person resources	4	7	1	0	0	0

^a^UF: University of Florida.

^b^VBC: virtual benefits counselor.

### Influence of Decision-Maker Factors on Preferred Health Information Source

We first present the results for the response variable of official versus nonofficial primary sources of information. The explanatory variables considered were age, health insurance use, and technology acceptance. However, the technology acceptance data did not show significant trends or results and are therefore not presented. The Cochran-Armitage results are presented in Table 7. For this response, all *P* values are less than .05, indicating that a trend exists between the explanatory variables and the use of an official primary source.

**Table 7:** Cochran-Armitage test results for official versus nonofficial primary sources of information response.

Explanatory variable	*z* value	Two-sided *P* value
Age (years)	5.657	<.001
Health insurance use	4.677	<.001
HILM^a^ understand terms	3.431	.001
HILM understand emergency payments	2.436	.01
HILM understand coverage	4.438	<.001
HILM finding out whether doctor is in-network	−3.503	.001

^a^HILM: Health Insurance Literacy Measure.

**Figure 8:**
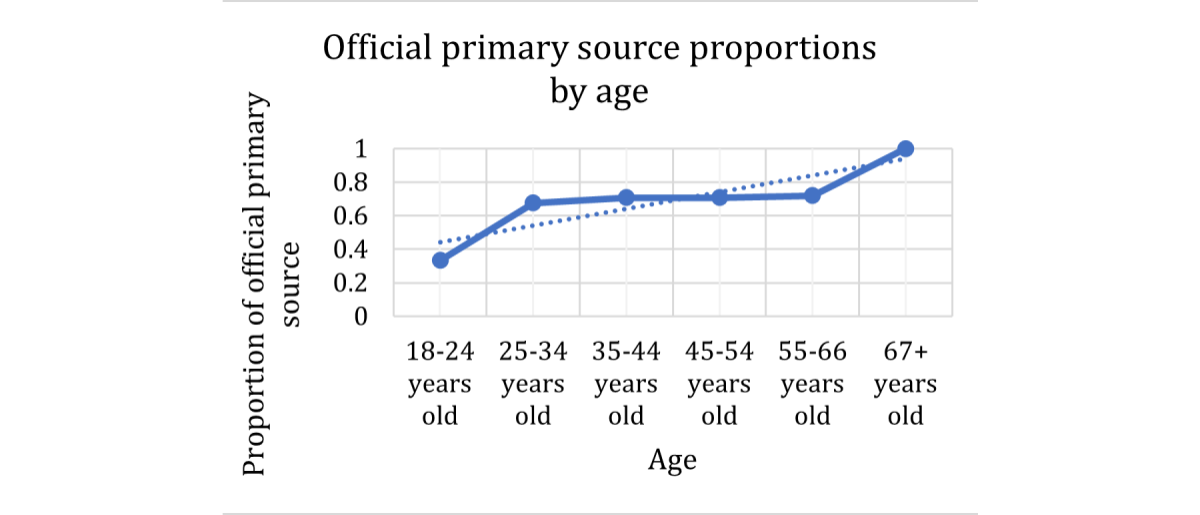
Contingency table proportions of official primary source per age group.

**Figure 9:**
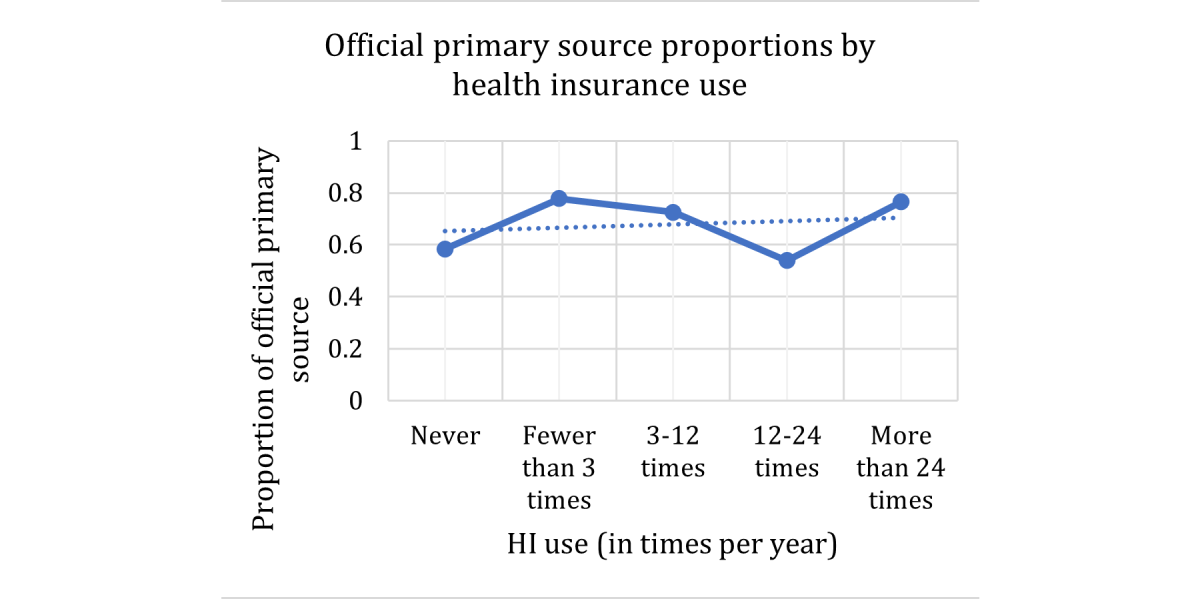
Contingency table proportions of official primary source per health insurance use group. HI: health insurance.

**Figure 10:**
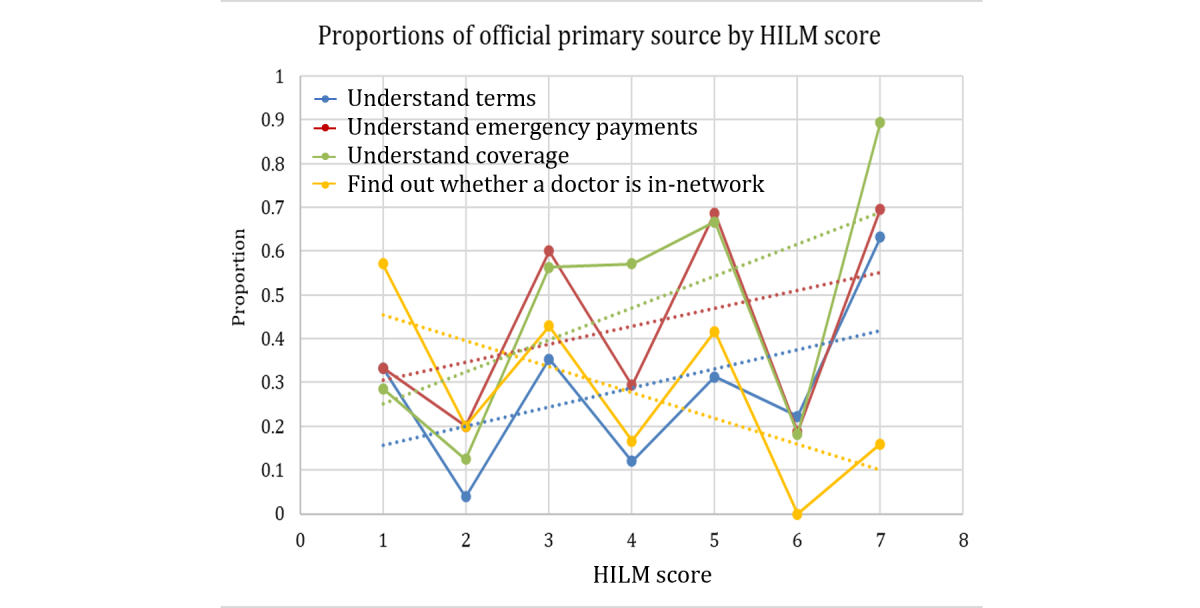
Contingency table proportions of official primary source per each Health Insurance Literacy Measure score. HILM: Health Insurance Literacy Measure.

These trends can be increasing linear trends, constant trends over time, or decreasing linear trends. The specific behavior for each explanatory variable is presented in the plots in Figures 8-10. In these plots, the proportions on the Y-axis refer to the proportions of participants who selected an official source as their primary source (true value in the contingency table). For the explanatory variable age, Figure 8 shows a significant increasing trend. The effect of age on this response slightly increases ordinally, which means that as age increases, the proportion of people who use official sources as primary sources increases as well. However, the middle-aged groups remained consistent at approximately 0.70. For the health insurance use explanatory variable in Figure 9, we see that the proportions are somewhat constant as the use of health insurance increases. As indicated in the *Methods* section, for the association between gender and the dependent variables, we used the φ coefficient of correlation. For this response of the official primary source, the coefficient was 0.03, which is extremely low and can be considered a negligible relationship.

The 4 plots for the HILMs are presented in Figure 10. The Cochran-Armitage test results showed that the proportion of people who use official primary sources is dependent on the scores of all 4 measures. The effect of the HILMs on the response variable changed ordinally. They all show increasing trends, except for the HILM finding out whether the doctor is in-network, for which the proportion of people who use official sources as primary sources decreases as the score increases.

**Figure 11:**
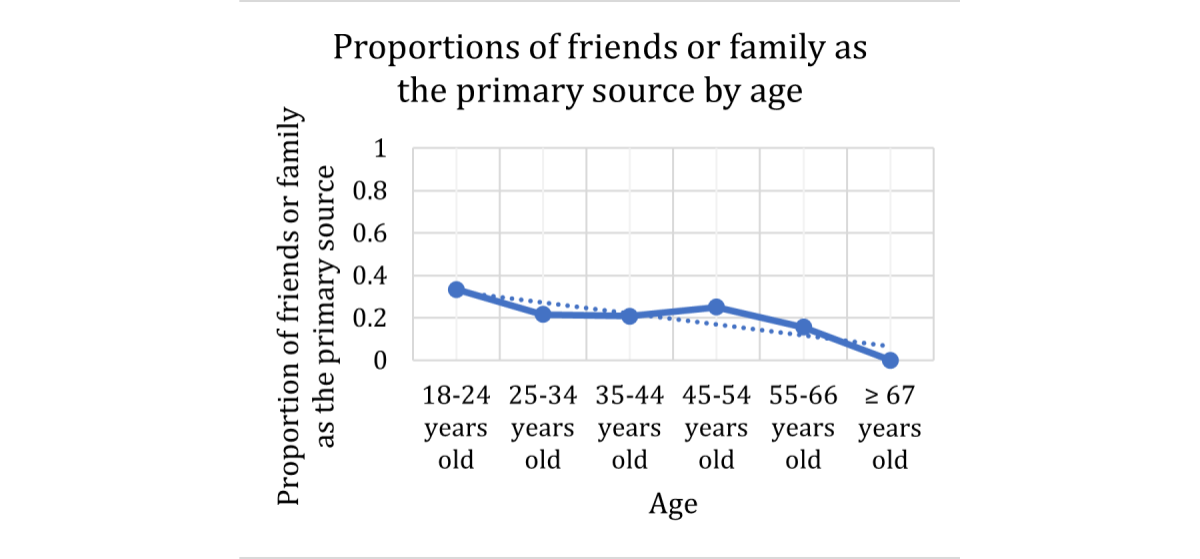
Contingency table proportions of friends or family as the primary source per age group.

**Figure 12:**
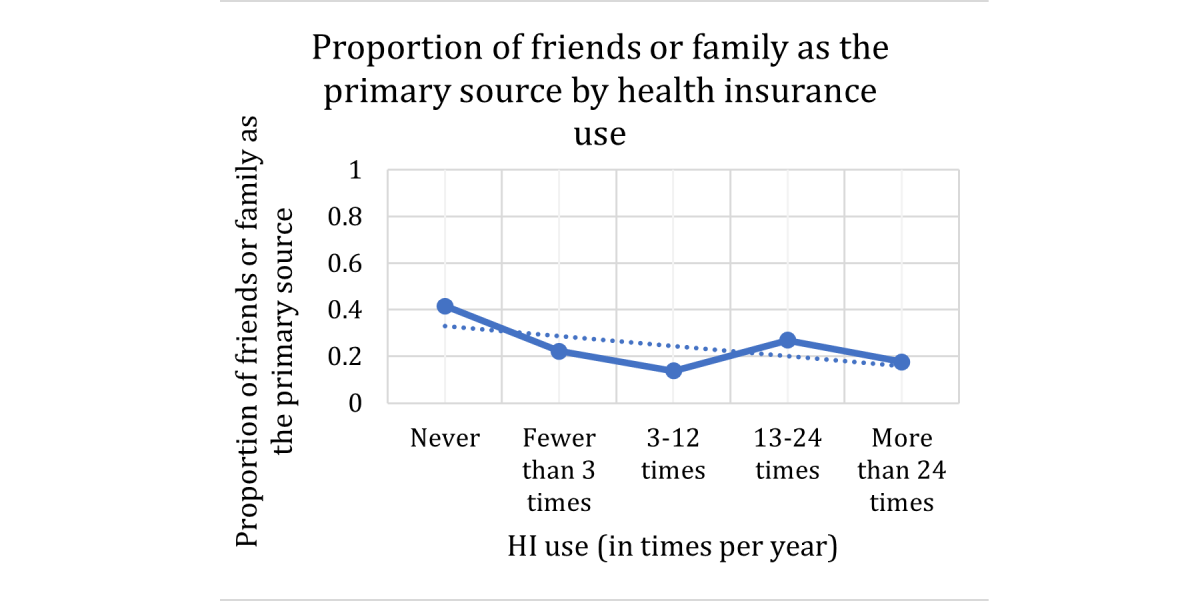
Contingency table proportions of friends or family as the primary source per health insurance use group. HI: health insurance.

**Figure 13:**
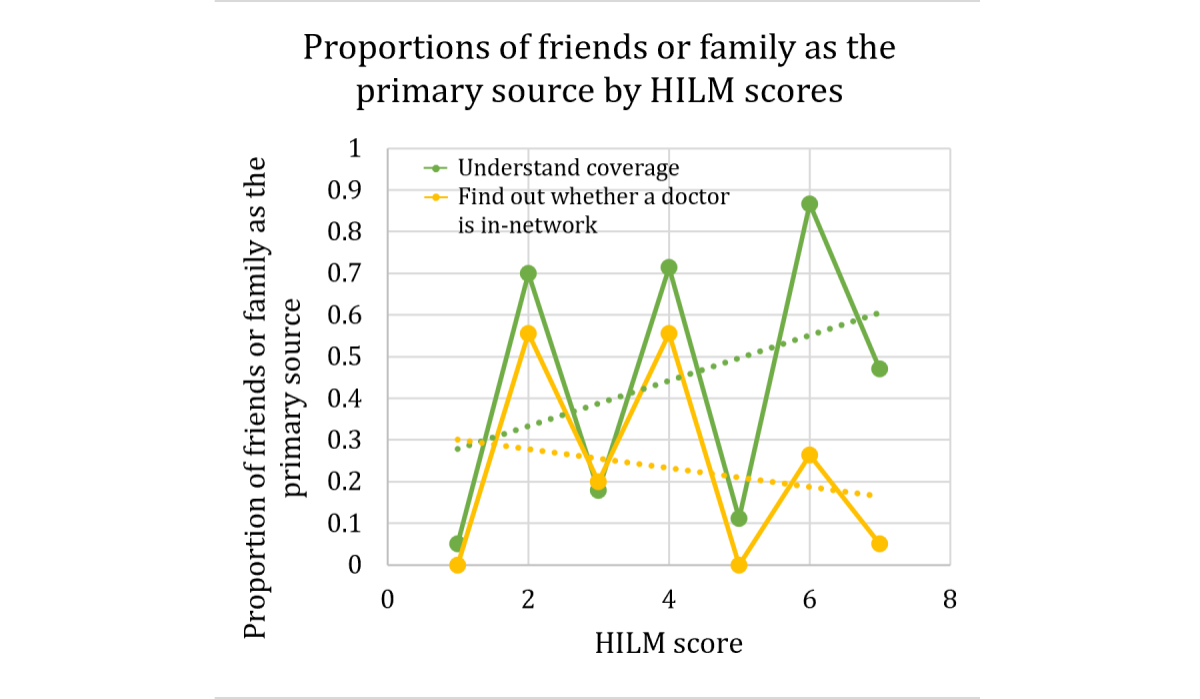
Contingency table proportions of friends or family as the primary source for 2 Health Insurance Literacy Measure scores. HILM: Health Insurance Literacy Measure.

For the response variable of friends or family versus not friends or family as a primary source of information, the Cochran-Armitage results are presented in Table 8 and the trend plots in Figures 8-10. In this case, the explanatory variables of age, health insurance use, HILM understand coverage, and HILM finding out whether the doctor is in-network had a *P*=.001, indicating that an association exists among these variables and the use of friends or family as primary sources. However, HILM understanding terms and HILM understand emergency payments do not indicate a significant relationship with the response. The specific behavior for the significant explanatory variables is presented in the plots in Figures 11-13. In these plots, the proportions on the Y-axis represent the proportions of participants who selected friends or family as their primary source of information (true value in the contingency table).

**Table 8:** Cochran-Armitage test results for friend or family vs non–friend or family responses.

Explanatory variable	*z* value	Two-sided *P* value
Age (years)	5.159	<.001
Health insurance use	3.451	.001
HILM^a^ understand terms	1.334	.18
HILM understand emergency payments	1.489	.14
HILM understand coverage	3.596	<.001
HILM finding out whether doctor is in-network	−3.225	.001

^a^HILM: Health Insurance Literacy Measure.

For the explanatory variable age, we now see in Figure 11 a slightly decreasing effect size for the friends or family primary source response. As age increases, the proportion of participants who make use of friends or family as a primary source decreases. Similarly, for health insurance use in Figure 12, we also see a slightly decreasing effect size as the use variable increases. Similar to the previous dependent variable, for this response of friends or family as a primary source, the φ coefficient for gender was 0.03. Once again, this is extremely low and can be considered a negligible relationship.

In terms of the HILMs, we only present the 2 measures that resulted in a significant association with this response. As mentioned previously, the 2 measures with significant relationships with friends and family as a primary source are HILM understand coverage (represented by the green lines in Figure 13) and HILM finding out whether the doctor is in-network (represented by the yellow lines in Figure 13). For HILM understanding coverage, we see an increasing trend, whereas for HILM finding out whether the doctor is in-network, we once again see a decreasing trend of proportions as the values of the scores increase.

## Discussion

### Principal Findings

Access to useful and accurate information is key to informed decision-making, especially for difficult decisions such as choosing a health insurance plan. Through the years, the health insurance environment has undergone many changes, and individuals have had to adapt their enrollment decision-making process accordingly. Therefore, we present a chronological background that evaluates the health plan information–seeking processes of individuals and the evolution of available sources and tools for health insurance information as an introduction to our work. In general, we learned that as the internet became more accessible, individuals mostly used digital and official sources of health information, as well as friends or family [24-28]. In more recent years, there have been greater efforts to include newer and varying tools within the sources of information. Given that little research has been conducted on the effect of these tools, it is important to study and understand the sources of information that users are currently relying on and the characteristics of users and health plans that contribute to the use of different sources, tools, and information types. With this survey, we take the initial steps toward answering these questions.

In terms of our sample, we had small groups in the youngest and older age categories, which was to be expected given our targeted population. Most of our participants used their insurance often (3-12 times per year), although the other use groups had balanced sample sizes. Overall, we saw medium to high health insurance literacy within our sample and high technology acceptance, which can also be a reflection of the targeted population. It was observed that the perceived usefulness of virtual agents among people who had used them multiple times was higher than among those who had only tried them. This could be because of selection bias (only those who liked using them or found them useful continued to do so) or because repeated interactions with virtual agents allowed people to find them more useful over time.

Our sample of employees from a large state university mainly used official employer or state websites as well as the official HR VBC Alex as sources of health insurance information, which are both digital and official sources. The previous results highlighted a dimension that seemed to differentiate the preferred source of health insurance information: whether the source of information was an official employer-provided source. The respondents who chose friends or family had a strong preference for this source, suggesting that there may be individual differences that led them to choose this unofficial source as their primary resource for health insurance information. The most prominent nonofficial source consisted of friends or family. Almost half of our sample relied on friends or family as part of their information-seeking strategy, and, interestingly, people who relied on friends or family tended to reach out to them first before considering other sources (27/58, 47% of the respondents who selected friends or family). In addition, our respondents mostly selected 2-3 sources of information, which means that within our sample, most people did not find all the information that they desired within a single source. This was to be expected and has been the trend since the early 2000s for general health information and health insurance information [24-28,38], especially observed in the study by Oetjen et al [31].

Our data analysis showed the following interesting trends. Looking at our first response, official versus nonofficial primary source, the proportion remains consistent at approximately 0.70 for the 3 middle-aged groups. For health insurance use, we observed a somewhat constant trend. These results were to be expected, given that official sources are suggested and encouraged by the employer’s HR department and contain the most specific plan information. For the HILM, we see an increasing trend (although not perfectly linear) across 3 of the 4 measures. Higher literacy is associated with increased use of official sources as the primary source. Given that higher literacy means that people understand and know more about health insurance in general, it makes sense that these people are more likely to better understand the details of the specific plans offered to them. Therefore, this type of specific information will be found in official sources. The fourth measure, which did not show an increasing trend, was the HILM finding out whether the doctor is in-network, which had a very right-skewed distribution, which might have caused such different behavior. Looking at our second response, the age variable showed a decreasing trend, indicating that younger people consider their friends or family as their primary source more than older people. We can imagine that younger people with less experience in plan enrollment might seek trusted advice when it comes to this important decision. For health insurance use, there was a slightly decreasing trend as well. This makes sense because more experienced insurance users are more familiar with how a plan works and, hence, would be less likely to seek trusted advice from friends or family. Finally, for the HILM, only 2 measures had significant associations, and both had behaviors similar to the official primary source response.

### Limitations

As suggested in the previous sections, our study included several limitations, which was to be expected given that this is an exploratory study. Allowing for snowball sampling to occur took power away from our data. Our skewed responses also contributed to this. The survey response rate was heavily skewed toward female respondents (103/126, 81.7%), resulting in small sample sizes for the other genders, which limited our ability to perform other analyses. This could be attributed to the fact that women are often the primary decision-makers for health concerns within a family [55] or because women are more likely to take the time to respond to health-related survey questions solicited through email and flyers. Future work should explore whether this finding continues to be robust health insurance decision-making, while also recruiting a larger sample to understand the health information–seeking behaviors of other genders. Our sample also had high technology acceptance and use, which also limits the generalizability of our findings.

In contrast, when studying the survey responses, we made note of possible areas of improvement in the way our questions were written, which will be considered when updating the survey for the next distribution. A limitation of this exploratory study concerned the factors explored in our questionnaire. The factors explored in this study (eg, age, household size, health insurance use, health insurance literacy, and technology adoption) were selected because they are most directly related to health insurance use and the methods used for accessing information; thus, they were factors that we hypothesized to have very strong connections to how individuals may seek sources of information. However, there are many demographic factors that can affect how individuals are able to access and understand different information sources that were not within the scope of this study, including race, numeracy, and health status. These factors should be explored in future work to understand both the systematic reasons for why certain types of health insurance sources are used and the underlying cognitive processes that help individuals understand and seek health insurance information. Another limitation and the most defining change to be made is the list of options for sources of health insurance information provided to the respondents. Our survey did not include sources such as extended networks, nor did it include the types of individual tools and decision aids. VBC Alex and the official websites considered in this study contain tools to support the decision-making process. For example, VBC Alex contains a cost estimator and comparison charts. These were not considered separately; therefore, we could not assess whether these aids are the drivers for the most selected sources.

In our next steps, we plan to extend our research to a more general population. We will create a new survey and work with the university’s Institute of Food and Agricultural Sciences (IFAS) extension office. The IFAS extension office is a partnership among state, federal, and county governments to provide scientific knowledge and expertise to the public. The IFAS has locations across the state and in every county. By working with the IFAS extension office, we will have access to community health workers who can help us reach a broader sample of the general population, regardless of employment status, for the new survey. This collaboration will result in access to a larger, more diverse sample with respect to race, income, and employment status. With a broader sample, we will have sufficient power to perform inferential statistics. In this new survey, the list of provided sources of information will be expanded and include more specific tools and decision aids to more effectively answer our research questions and address some of the limitations identified in this study. We expect some of our findings to extend to a more general population. Zhao et al [46], who used a more general population, provided the closest comparison to our study; however, the authors focused on the evaluation of a specific web-based decision tool—the SMHP tool—whereas the aim of our study is to understand the sources of information for health insurance plan selection. Nevertheless, there are some common overlaps. First, Zhao et al [46] also reported a high number of female respondents or participants. Second, they found that high-income populations were more likely to seek web-based information to support their health insurance plan decision-making process. As our inclusion criteria required the participants to be employed full-time, we assumed that our population has a higher income than the general population, and thus the responses in our study are likely biased toward official web-based sources of health insurance information. Finally, we expect that among the general population, participants with high HILM scores will still prefer official primary sources of health insurance information. In addition, in our next steps, we will collect data on the outcome of the enrollment decision and examine relationships between the sources of information (and the number of sources) used and the final enrollment decision.

## Appendix

Multimedia Appendix 1 Health Insurance Information Sources Survey.

Multimedia Appendix 2 Graph of the ranking occurrences per source. UF: University of Florida, VBC: virtual benefits counselor.

## Abbreviations

HIISS: Health Insurance Information Sources Survey
HILM: Health Insurance Literacy Measure
HR: human resources
IFAS: Institute of Food and Agricultural Sciences
SMHP: Show Me My Health Plans
VBC: virtual benefits counselor
