# Supplementary material for: Informed Decision-making for Health Insurance Enrollment: Survey Study
Source: JMIR Form Res. 2021 Aug 12;5(8):e27477. doi: 10.2196/27477 (PMC8391737; doi:10.2196/27477)
Supplement: Multimedia Appendix 1 [file formative_v5i8e27477_app1.docx]

Health Insurance Information Sources Questionnaire

Start of Block: Consent

**** Research Participant   Informed Consent Form***

* Please read this document carefully before you decide to participate in this research study. **Your participation is voluntary, and you can decline to participate, or withdraw consent at any time, with no consequences**.

*** Study Title:**   Health Insurance Literacy and Decision-Making Using Digital Decision-Aids

*** Person(s) conducting the research:**

PI: Assistant Professor, Department of Industrial and Systems Engineering
Co-PI: Assistant Professor, Department of Industrial and Systems Engineering

*** Purpose of the research study:**   Choosing a health insurance plan is a difficult and complex decision with potentially large financial- and health-related consequences. Only 4% of the U.S. population accurately understands basic health insurance terminologies; thus, decision-aids are critical to helping individuals understand health insurance plan options for informed decision-making. This study will help us understand where you look for information when you need to make health insurance enrollment decisions as an employee at a large state university.

* **What you will be asked to do in the study:**   You will be asked to answer a short questionnaire. We will collect information on age, gender, marital status, number of dependents, hiring year, income level, technology acceptance, role in healthcare decision-making within the household, sources of digital information for health insurance enrollment, and a ranking of the sources used. In addition, if you are interested in participating in the follow-up user study that pays $50, you may provide your name, phone and e-mail.

*** Time required:**   We expect this survey to take approximately 15 minutes.

*** Risks and benefits:**   There are no anticipated risks to you.  One benefit of this this research is that it can lead to improved health insurance decision-aids that help individuals make better health insurance and health care decisions.

*** Confidentiality:**   If you agree to participant in the study, any personal information we may collect during the experiment will be stored in a locked cabinet or password-protected computer which is accessible only to members of the research team. No personal information will be published. All data that we collect from you will be assigned a non-descriptive alias.    *** Compensation:**   You will receive no compensation for your participation in this online survey. However, you may qualify for a paid follow-up study.

*** Withdrawal from the study:**   * You are free to withdraw your consent and to stop participating in this study at any time without consequence.  You may skip any question that you don't wish to answer, and you will not be asked to disclose any medical conditions or other health-related information.   You may decide not to continue in the research at any time without it being held against you. The person in charge of the research can remove you from the research at any time without your approval for any reason. Possible reasons for removal include: Inability to follow study directions, unsolvable technical difficulty during data collection, or the discovery of existing conditions that would exclude you from participating in the study.   * If you wish to discuss the information above or any discomforts you may experience, please ask questions now or contact one of the research team members listed at the top of this form.   * If you have any questions regarding your rights as a research subject, please contact the Institutional Review Board (IRB02) office (.)

*** Agreement:**   Now that you’ve read about the study, if you wish to participate, click the “I agree to participate” button to continue; if you do not consent to participate, click “I do not wish to participate” or just close this window.

- Yes, I agree to participate.
- No, I do not agree to participate.

End of Block: Consent

Are you a current part-time or full-time employee (Faculty, Clinical Faculty, TEAMS or OPS) of this university?

- Yes
- No

Your current employment status is:

- 1.0 FTE (Full Time Employment)
- 0.75 FTE (30 hours per week)
- Less than 0.75 FTE

 For your last enrollment period,  were you responsible, or did you share responsibility, in making health insurance decisions within your household?

- Yes, I was primarily responsible for health insurance decisions in my household.
- Yes, I shared responsibility for health insurance decisions in my household.
- No, I did not share responsibility for health insurance decision in my household.

 For your last enrollment period,  how active were you in making health insurance decisions within your household?

|  | 1 | 2 | 3 | 4 | 5 |
| --- | --- | --- | --- | --- | --- |
| Making health insurance decision |  |  |  |  |  |

**Demographic Questions**

What is your age?

- 18-24 years old
- 25-34 years old
- 35-44 years old
- 45-54 years old
- 55-66 years old
- 67 plus years old
- Prefer not to say

Which of these best describes your gender?

- Male
- Female
- Other
- Prefer not to say

Which of these best describes your marital status?

- Single
- Married or domestic partnership
- Prefer not to say

Do you have dependents beside a spouse or a partner?

- Yes
- No
- Prefer not to say

How large is your household?

- 1 to 2
- 3 to 5
- 6 or more

When were you hired at this university?

- Less than 1 year ago
- Between 2-5 years ago
- More than 5 years ago

End of Block: Demographic Questions

How many different health insurance plans are in your household?

- 0
- 1
- 2
- 3 or more

How many people aside from yourself are covered in your health insurance?

- 0
- 1
- 2 to 4
- 5 or more

Do you get your health insurance through this university?

- Yes
- No
- Prefer not to say

How often do you or your dependents use your health insurance?

- Never
- Less than 3 times a year
- 3 to 12 times a year
- 13 to 24 times a year
- More than 24 times a year
- Prefer not to say

Has your knowledge in health insurance increased because of a major event that happened to you or someone you know (other than a change in a employment)?

- Yes
- No
- Prefer not to say

**Sources of Information**

Where do you find information about health insurance plans? (Select all that apply)

- Official Human Resources's Alex, an online, virtual benefits counselor
- Official employer or state of website
- Other online websites or resources (e.g. Google, healthcare.gov; please indicate the website or resource) ________________________________________________
- Official Human Resource's in-person benefits counselors
- Friends or family
- Other in-person resources (please indicate the resources) ________________________________________________

|  |  |
| --- | --- |

Please rank the sources of information that you use for health insurance enrollment decisions from most important (1) to least important. (Please select one ranking per source)

______ Official Human Resources's Alex, an online, virtual benefits counselor

______ Other official employer or state website

______ Other online websites or resources (e.g. Google, healthcare.gov; please indicate the website or resource)

______ Official Human Resource's in-person benefits counselors

______ Friends or family

______ Other in-person resources (please indicate the resources)

**Health Insurace Literacy Measures**

How confident would you feel that you understand health insurance terms (e.g., co-pay, deductible, co-insurance, premium)?

|  | 1 | 2 | 3 | 4 | 5 | 6 | 7 |
| --- | --- | --- | --- | --- | --- | --- | --- |
| Understanding of health insurance terms |  |  |  |  |  |  |  |

When comparing health plans, how confident are you in understanding what needs to be paid for emergency department visits?

|  | 1 | 2 | 3 | 4 | 5 | 6 | 7 |
| --- | --- | --- | --- | --- | --- | --- | --- |
| Understanding emergency visit payments |  |  |  |  |  |  |  |

How confident are you in knowing what is and is not covered before you receive a health care service?

|  | 1 | 2 | 3 | 4 | 5 | 6 | 7 |
| --- | --- | --- | --- | --- | --- | --- | --- |
| Coverage knowledge |  |  |  |  |  |  |  |

When using your health insurance plan, how likely are you to find out if a doctor is in-network before you see him/her?

|  | 1 | 2 | 3 | 4 | 5 | 6 | 7 |
| --- | --- | --- | --- | --- | --- | --- | --- |
| Finding in-network doctors |  |  |  |  |  |  |  |

**Technology Acceptance Questions**

On a scale of 1 to 10, with  1 being “very inexperienced” and 10 being “very experienced” how would you rate your level of experience with technology (e.g. cell phones, automatic teller machines, digital cameras, computers, etc.)?

|  | 1 | 2 | 3 | 4 | 5 | 6 | 7 | 8 | 9 | 10 |
| --- | --- | --- | --- | --- | --- | --- | --- | --- | --- | --- |
| Level of experience with technology |  |  |  |  |  |  |  |  |  |  |

Some people prefer to avoid new technologies as long as possible while others like to try them out as soon as they become available. In general, how would you rate yourself as being an avoider or an early adopter of new technology?

|  | 1 | 2 | 3 | 4 | 5 | 6 | 7 | 8 | 9 | 10 |
| --- | --- | --- | --- | --- | --- | --- | --- | --- | --- | --- |
| Adoption of new technology |  |  |  |  |  |  |  |  |  |  |

How would you rate your overall level of trust in technology?

|  | 1 | 2 | 3 | 4 | 5 | 6 | 7 | 8 | 9 | 10 |
| --- | --- | --- | --- | --- | --- | --- | --- | --- | --- | --- |
| Trust in technology |  |  |  |  |  |  |  |  |  |  |

Have you ever interacted with a “virtual agent”, “chatbot”, or “virtual rep” when interacting with a website or web service? Virtual agents provide automated customer service using a conversational interface.

- Yes, multiples times.
- Yes, I have tried them.
- No, I have not tried them.
- Not sure

On a scale of 1 to 10, with 1 being “not useful at all” and 10 being “extremely useful”, how would you rate the **usefulness** of the virtual agents you’ve interacted with in the past?

|  | 1 | 2 | 3 | 4 | 5 | 6 | 7 | 8 | 9 | 10 |
| --- | --- | --- | --- | --- | --- | --- | --- | --- | --- | --- |
| Usefulness of virtual agents |  |  |  |  |  |  |  |  |  |  |

On a scale of 1 to 10, with 1 being “not easy to use at all” and 10 being “extremely easy to use”, how would you rate the **ease of using** the virtual agents you’ve interacted with in the past?

|  | 1 | 2 | 3 | 4 | 5 | 6 | 7 | 8 | 9 | 10 |
| --- | --- | --- | --- | --- | --- | --- | --- | --- | --- | --- |
| Ease of using the virtual agents |  |  |  |  |  |  |  |  |  |  |

**Experimental Study**

If you qualify, are you willing to participate in a follow-up study on health insurance decision making that will be conducted in person on the university campus? This in person study will pay $50 and take approximately 90 minutes.

- Yes
- No

If so, please enter an e-mail address  that we may contact you at.

________________________________________________________________

Please enter your first and last name.

________________________________________________________________
