# Supplementary figures and images for: Informed Decision-making for Health Insurance Enrollment: Survey Study
Source: JMIR Form Res. 2021 Aug 12;5(8):e27477. doi: 10.2196/27477 (PMC8391737; doi:10.2196/27477)

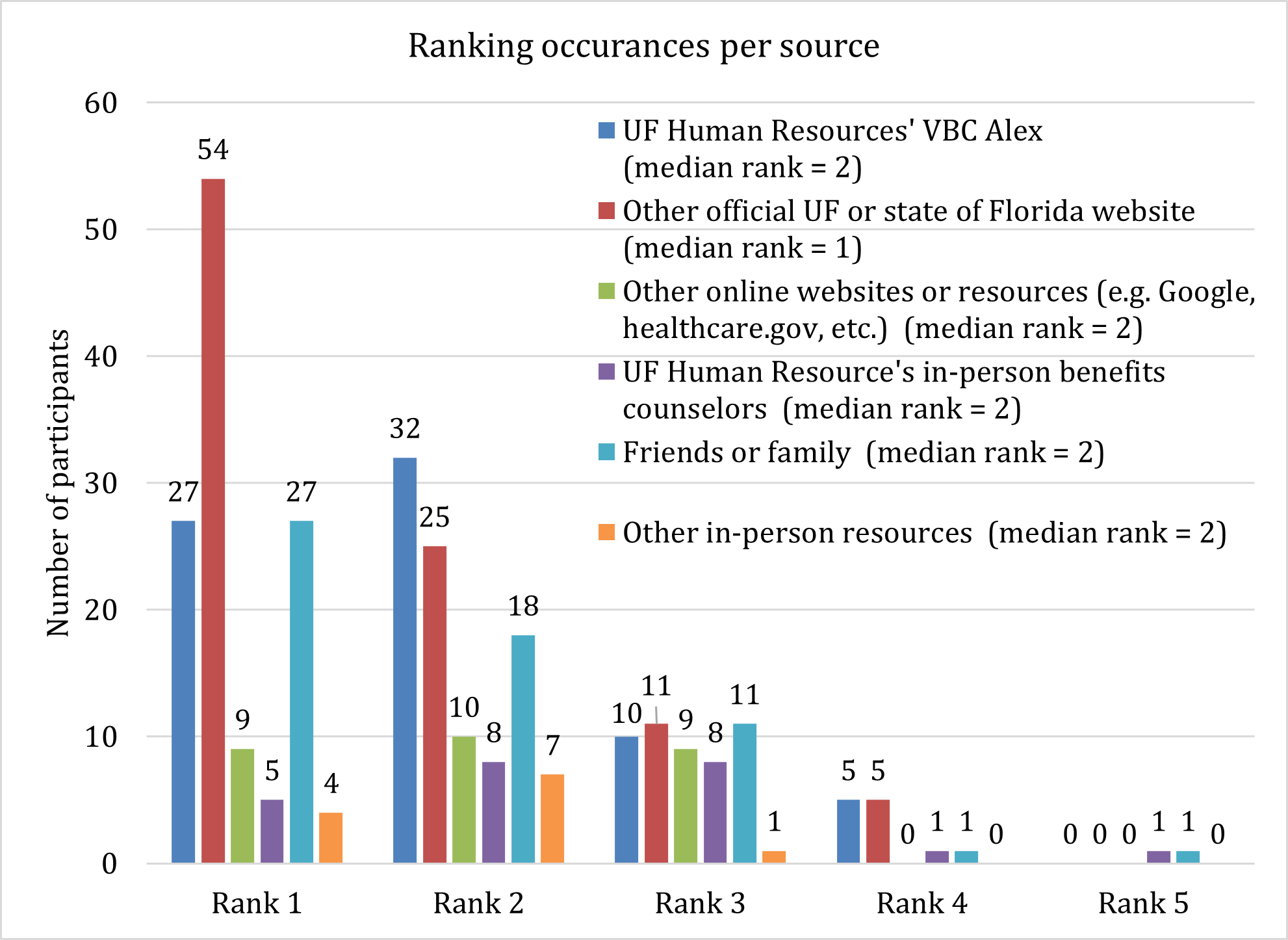

Supplement: Multimedia Appendix 2 [file formative_v5i8e27477_app2.png]
